# Synchronous squamous cell carcinoma and diffuse large B-cell lymphoma of the head and neck: the odd couple

**DOI:** 10.1259/bjrcr.20150271

**Published:** 2016-01-19

**Authors:** Ann C Raldow, Johann G Brown, Nicole Chau, Matthew S Davids, Danielle N Margalit, Roy B Tishler, Andrea Ng, Jonathan D Schoenfeld

**Affiliations:** ^1^ Harvard Radiation Oncology Program, Boston, MA, USA; ^2^ Department of Radiation Oncology, Brigham and Women’s Hospital/Dana-Farber Cancer Institute, Boston, MA, USA; ^3^ Department of Medical Oncology, Brigham and Women’s Hospital/Dana-Farber Cancer Institute, Boston, MA, USA

## Abstract

We report the case of an 81-year-old male with synchronous recurrent cutaneous squamous cell carcinoma (SCC) metastatic to the parotid and diffuse large B-cell lymphoma of the head and neck. These malignancies necessitated integrated multidisciplinary treatment within a short time period. Superficial parotidectomy was followed by chemotherapy for lymphoma. The subsequent radiation treatment plan combined both sequential boost to treat the SCC surgical bed to a higher dose compared with the lymphoma, and dose-painting intensity-modulated radiation therapy (IMRT) to differentially dose the areas at risk. The treatment was tolerated well. The restaging scans demonstrated no evidence of either lymphoma or SCC. This case highlights the importance of combined modality treatment for two concurrent aggressive malignancies in the head and neck region. Radiation treatment planning incorporated both sequential boost and dose-painting IMRT to integrate comprehensive treatment for both malignancies.

## Introduction

Diffuse large B-cell lymphoma (DLBCL) is the most prevalent non-Hodgkin lymphoma histology, representing approximately 30% of new cases.^1^ The established treatment guidelines for early-stage DLBCL include chemotherapy followed by radiation to 30–36 Gy in 2 Gy fractions.^2^ Involved nodal or site radiation fields have recently replaced the more extensive involved field radiation portals, allowing for greater sparing of the surrounding normal tissues.^3^

Cutaneous squamous cell carcinoma (SCC) is the second most frequent type of skin cancer histology and one of the most common malignancies in the USA.^4^ Because cutaneous SCC is not routinely reported to cancer registries, its exact incidence is unknown. The 5-year overall survival rate for patients with lymph node metastases is approximately 50–60%.^5^ Once SCC has spread to regional lymph nodes, surgery plus adjuvant radiation therapy (RT) with or without concurrent chemotherapy is the widely accepted treatment.^6^ Post-operative doses for resected SCC typically range from 45 to 66 Gy, and although electron treatment is often utilized for primary site skin cancer treatment, dose-painting intensity-modulated radiation therapy (IMRT) can be used in the head and neck region to differentially dose areas of high, intermediate and low risk, while sparing adjacent normal structures.^6^

Herein, we report a case of an 81-year-old male patient diagnosed with SCC of the skin metastatic to an intraparotid lymph node, who, immediately after surgery for this malignancy, was also diagnosed with a rapidly progressing Stage IIAE DLBCL of the head and neck region. To our knowledge, this case represents the first documented presentation of synchronous SCC of the skin recurrent to the lymph node and DLBCL of the head and neck. This unique presentation necessitated a strategy that integrated treatment for both malignancies within a relatively short time period and highlights the importance of multidisciplinary care. This presentation also required a radiation treatment plan that was able to concurrently treat areas in close proximity with doses and fractionation appropriate for both lymphoma and recurrent SCC.

## Clinical presentation, imaging findings and treatment

**Figure 1.**
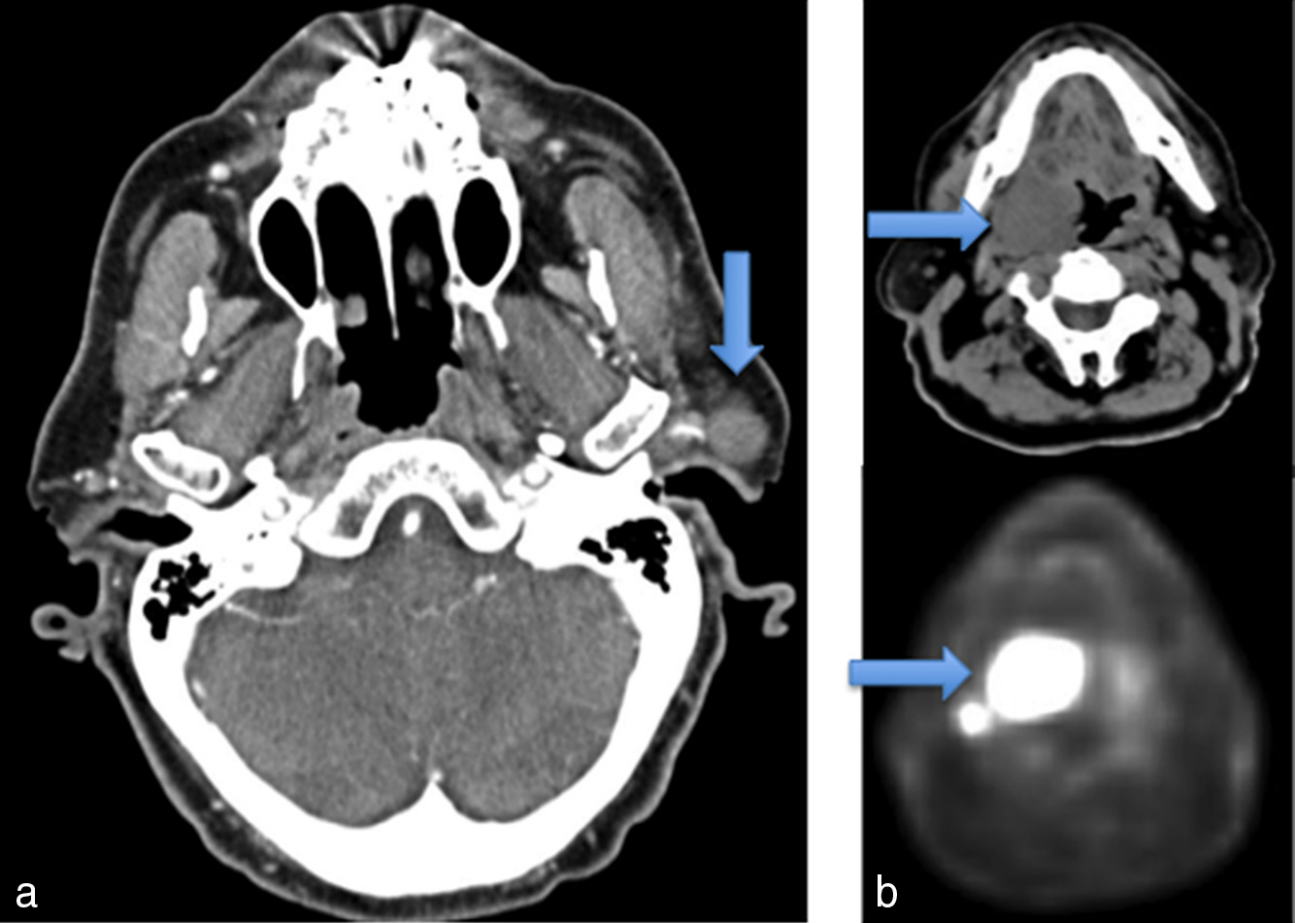
(a) A pre-auricular mass representing a 1.9-cm sarcomatoid squamous cell carcinoma within an intraparotid lymph node (arrow). (b) A fludeoxyglucose-avid right tonsillar mass extending to the base of the tongue (arrows) was biopsied to reveal diffuse large B-cell lymphoma.

Our patient was an 81-year-old male with a history of multiple prior skin cancers and cardiac comorbidities, initially treated for sarcomatoid SCC of the left scalp at an outside institution. The initial wide local excision with rotation flap closure revealed skin cancer invasive to a depth of 8 mm with perineural but no lymphovascular invasion. The pathological margins were negative. 9 months later, the patient developed a pre-auricular mass (Figure 1a). Initial attempts at biopsy were inconclusive, and therefore, a left superficial parotidectomy with facial nerve dissection was performed, revealing a 1.9-cm sarcomatoid SCC within an intraparotid lymph node, likely metastatic from the patient’s prior skin cancer. The closest margin was less than 1 mm, and the MRI did not demonstrate any evidence of gross residual disease.

In the postoperative recovery period, our patient developed an increasingly sore throat and dysphagia. An examination revealed a large contralateral right tonsillar mass extending beyond the glossotonsillar sulcus to the base of the tongue that was biopsied to reveal DLBCL. Specifically, it showed tumour cells with large fascicular nuclei and scant cytoplasm that stained strongly positive for CD20 and CD45. The cells were negative for CD3, CD30, cytokeratins, cytokeratin CAM 5.2 and AE13, and melanoma markers S100, human melanoma black 45 and melanoma-associated antigen recognized by T cells 1. *MYC* rearrangement was not detected by fluorescent *in situ* hybridization. A staging positron emission tomography (PET)-CT scan demonstrated a fludeoxyglucose (FDG)-avid mass measuring at least 4 cm, centred about the right oropharynx and exerting a significant mass effect upon the oropharyngeal airway (Figure 1b). There were also two small FDG-avid nodes in the posterior right mandibular region.

Our patient met with a multidisciplinary treatment team specializing in both lymphoma and head and neck cancers to discuss treatment options. The patient favoured an aggressive approach that would maximize his chances of eradicating both malignancies. The patient’s untreated lymphoma posed an immediate threat to the airway, and therefore, the patient was urgently started on chemotherapy with three cycles of rituximab, cyclophosphamide, doxorubicin, vincristine and prednisone (R-CHOP). Three cycles of R-CHOP followed by RT is considered standard of care as per the National Comprehensive Cancer Network guidelines, and this approach was chosen given the patient’s age and comorbidities.^2^ This treatment paradigm had achieved favourable results in the Southwest Oncology Group study 0014.^7^ The patient had improvement of his symptoms shortly after initiating therapy.

A restaging PET-CT scan performed after chemotherapy revealed the DLBCL in the right neck had a complete response to chemotherapy, and there was no recurrent SCC identified in the parotid bed or the left neck. The patient was therefore treated with adjuvant radiation to the involved site (ISRT) for the DLBCL and parotid bed and left neck for the recurrent SCC. ISRT for DLBCL targets the sites of the originally involved lymph nodes and extranodal disease (if any) prior to any therapy and spares the adjacent healthy tissues.^2^

**Figure 2.**
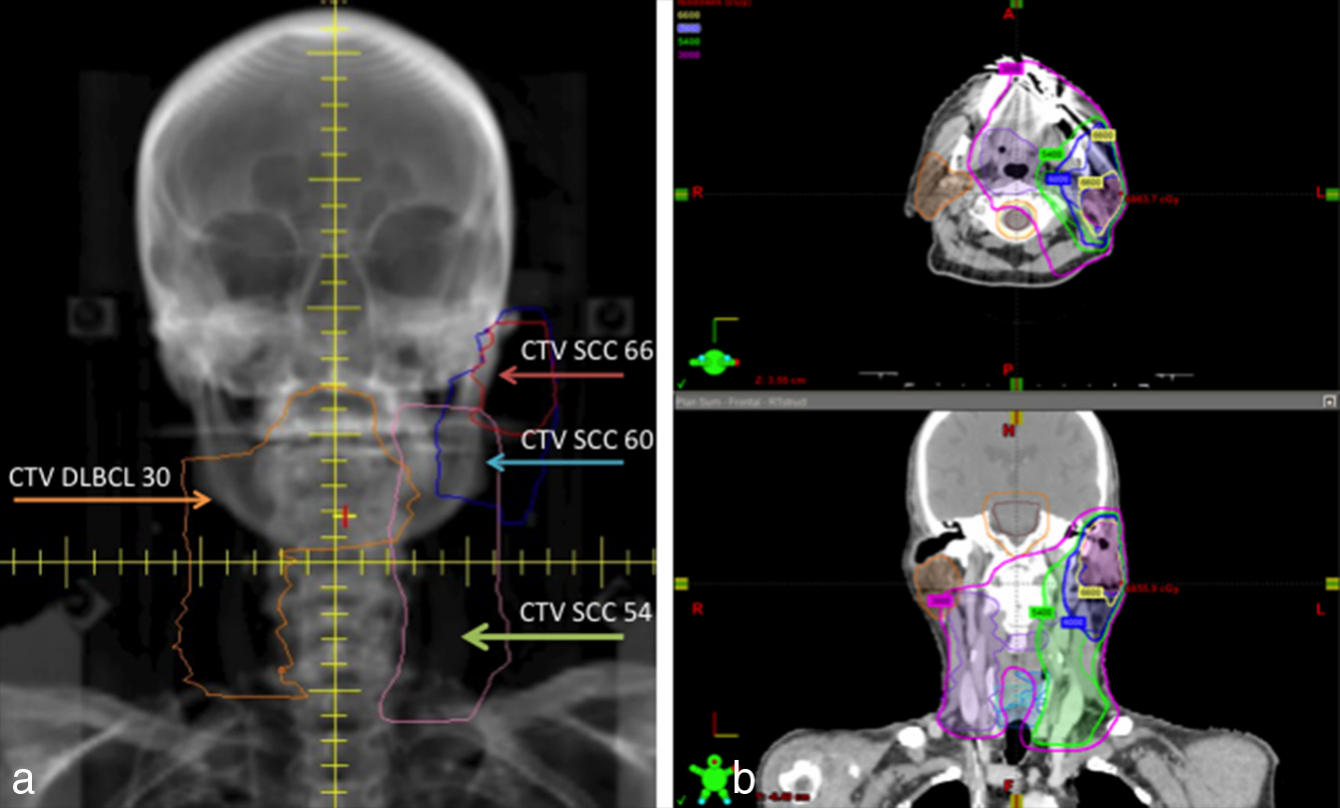
Cumulative radiation plan for a patient with synchronous DLBCL and recurrent SCC utilizing both sequential cone-down and dose-painting intensity-modulated radiation therapy to deliver involved site radiation to the lymphoma and post-operative radiation to areas at risk for SCC recurrence. (a) Digitally reconstructed radiograph with delineated target volumes: volume corresponding to areas involved by DLBCL before chemotherapy (DLBCL CTV 30), and post-operative high-, intermediate- and low-risk volumes corresponding to the resected SCC (SCC CTV 66, 60 and 54). Clinical target volumes were expanded by 5 mm to create PTVs. (b) Isodose lines demonstrating doses delivered to areas at risk in axial (top) and coronal (bottom) views. DLBCL, diffuse large B-cell lymphoma; PTV, planning target volume; SCC, squamous cell carcinoma.

The treatment plan utilized both sequential cone-down (a radiation boost to a reduced treatment volume) and dose-painting IMRT. The initial plan was delivered in 15 fractions and treated the lymphoma field to a total dose of 30 Gy. The left parotid bed and neck were treated with 33, 30 and 27 Gy to areas of high-, intermediate- and low-risk disease, respectively. The 15-fraction second course did not provide any additional treatment to the lymphoma field, but brought the total dose delivered to the left parotid bed and neck to 66, 60 and 54 Gy to areas of high-, intermediate- and low-risk disease, respectively (Figure 2).

The radiation treatment was tolerated well. A percutaneous endoscopic gastrostomy (PEG) tube was placed to supplement oral intake before starting RT, but the dysphagia resolved shortly after completion of the therapy and the PEG tube was removed 2 months following the end of radiation. The restaging scans 4 months after the completion of all treatment demonstrated no evidence of either lymphoma or SCC.

## Discussion

Both DLBCL and SCC of the skin are more common in elderly patients; however, the occurrence of recurrent cutaneous SCC metastatic to regional lymph nodes and DLBCL concurrently in the same anatomic region is an unlikely event that presents significant treatment challenges. Indeed, lymph node metastases occur in less than 5% of cutaneous SCCs^8^ and DLBCL of the head and neck accounts for less than 15% of all DLBCL cases.^9^ RT is a frequently used treatment for both localized DLBCL and SCC of the head and neck metastatic to the lymph nodes. The patient’s DLBCL was growing rapidly at the time of presentation and therefore necessitated immediate treatment following the superficial parotidectomy for the SCC. Given the patient’s age, his favourable prognostic features,^10^ and the need for adjuvant treatment to the left parotid, a decision was made to plan for three cycles of chemotherapy followed by ISRT.^11^

Our patient was also diagnosed with recurrent sarcomatoid SCC of the head and neck metastatic to a parotid lymph node. Sarcomatoid SCC is an aggressive squamous cell variant that may be more likely to recur following treatment;^12^ indeed, because of multiple high-risk features, including perineural invasion, we would have initially considered adjuvant treatment to the resection bed and regional lymph nodes had the patient been treated at our centre prior to recurrence. Adjuvant RT is typically recommended for recurrent SCC of the head and neck and for patients with involved parotid lymph nodes. In our patient, concurrent radiosensitizing chemotherapy was considered, but ultimately deferred because of the patient’s age, comorbidities and recent systemic lymphoma treatment.

Our challenges when developing a radiation treatment plan were: (1) the significantly different doses that needed to be administered to the right and left neck: 54–66 Gy to the left parotid bed/neck and 30 Gy to the right oropharynx/neck involved by lymphoma; and (2) the desire to keep fraction sizes above 180 cGy per fraction. Sequentially treating both regions was considered, but would have been associated with an excessive delay in treating the second region. Therefore, our radiation treatment plan needed to start out by treating all areas at risk concurrently.

Radiation treatment plans that include sequential cone-downs, where the original treatment area is reduced in size to enable continuous dose delivery while sparing the surrounding normal tissues, have a long history in head and neck treatment planning and are generally used to boost areas of high-risk or gross disease.^13^ However, in the recent years, this technique has largely been replaced by dose-painting IMRT, which allows for differential dosing of multiple regions within a single treatment plan. In our case, a one-course IMRT plan with dose-painting was unacceptable, as the lymphoma volume needed to be treated in 2 Gy fractions, resulting in fractions as high as 4.4 Gy delivered to areas at highest risk for SCC. In contrast, a radiation treatment plan consisting entirely of sequential cone-downs would have required co-ordinating three separate plans, potentially leading to unacceptable hot spots or high doses to organs at risk when the doses from these plans were combined.

Therefore, our final treatment strategy combined both sequential boost to treat the SCC with a higher dose compared with the lymphoma, and dose-painting IMRT to differentially dose the areas at risk within the operative bed and the left neck. Four treatment volumes [with 5 mm expansion of clinical target volume to planning target volume (PTV)] were delineated: an ISRT volume corresponding to areas involved by DLBCL before chemotherapy (DLBCL PTV 30), and post-operative high-, intermediate- and low-risk areas corresponding to the resected SCC (SCC PTV 66, 60 and 54, respectively). The first course used 10 IMRT fields consisting of 15 fractions. The SCC PTV 66 received 220 cGy, the DLBCL PTV 30 and SCC PTV 60 received 200 cGy, and the SCC PTV 54 received 180 cGy per fraction, for total doses of 33 , 30 and 27 Gy, respectively. The second course used seven fields consisting of a cone-down volume that excluded the DLBCL PTV. The second course comprised 15 fractions and brought the total dose to 66, 60 and 54 Gy to areas of high-, intermediate- and low-risk disease, respectively, in the left parotid bed and neck. The areas continued to receive the same dose fractionation as in the first course: 220 , 200 and 180 cGy per fraction for SCC PTV 66, 60 and 54, respectively.

Several points deserve further discussion. First, a modestly hypofractionated daily fraction size of 220 cGy was used in the absence of chemotherapy to treat the area of closest margin in the left parotid bed given the risk of occult microscopic positive margin. An integrated boost of 66 Gy delivered in 30 fractions is currently being used in the Radiation Therapy Oncology Group study 0216, where post-operative regions felt to be at particularly high risk for recurrence may be defined at the discretion of the radiation oncologist. Second, by using seven fields for the cone-down volume, with almost all the beams entering the patient from the left side, the midline and right-sided organs at risk were spared to a large degree. In fact, the combination of dose-painting and cone-down were able to spare the adjacent normal structures and, in particular, the right parotid gland to a significant degree (Table 1). This degree of normal tissue sparing would not have been possible without IMRT.

**Table 1.** Cumulative plan percentages of PTV receiving prescription dose and doses to critical structures.

Structures	Achieved
SCC PTV 66	96.7%
SCC PTV 60	95.2%
SCC PTV 54	98.8%
Diffuse large B-cell lymphoma PTV 30	99.9%
Spinal cord	29 Gy (max)
Spinal cord plus 5 mm	34.9 Gy (max)
Spinal cord plus 7 mm	39 Gy (max)
Brainstem	30.6 Gy (max)
Brainstem plus 7 mm	36.2 Gy (max)
Parotid, right	18.6 Gy (mean) V30 = 11.7%
Cochlea, right	8.2 Gy (max)
Cochlea, left	27.8 Gy (max)
Oesophagus	14 Gy (max)
Larynx	28.5 Gy (max)
Submandibular gland, right	34.5 Gy (max)
Postcricoid	27.1 Gy (max)

Max, maximum; PTV, planning target volume; SCC, squamous cell carcinoma.

The synchronous presentation of DLBCL involving the oropharynx and sarcomatoid SCC metastatic to the parotid is unique and required care by a multidisciplinary team. The final radiation treatment plan combined treatment planning techniques that have become widespread in modern radiation oncology practice. Pre-treatment imaging was used to design custom treatment fields based on the pre-chemotherapy lymphoma tumour volume and pre-operative extent of metastatic SCC. A sequential boost was used to treat the lymphoma and the SCC treatment volumes to significantly different doses without delaying the treatment for either malignancy. Dose-painting IMRT was used to differentially dose areas at risk, while minimizing dose to the adjacent normal structures.

## Learning points

1. This case represents the first documented presentation of synchronous SCC of the skin recurrent to the lymph nodes and DLBCL of the head and neck.
2. A multidisciplinary treatment strategy, including surgery, chemotherapy and radiation, integrated treatment for the two malignancies within a relatively short time period.
3. Our radiation treatment plan incorporated both sequential radiation boost and IMRT with dose-painting in order to concurrently treat areas of the head and neck with radically different doses, while keeping fraction sizes appropriate and maintaining normal tissue sparing.
4. We highlight the use of RT in the adjuvant setting following surgery for cutaneous SCC and following chemotherapy for DLBCL; in both cases, reconstruction of the pre-treatment volumes were important for target delineation.

## Consent

Informed consent to publish (including images and data) was obtained and is held on record.
